# The “StemDif Sensor Test”: A Straightforward, Non-Invasive Assay to Characterize the Secreted Stemness and/or Differentiation Activities of Tumor-Derived Cancer Cell Lines

**DOI:** 10.3390/biomedicines11123293

**Published:** 2023-12-13

**Authors:** Aya Abou Hammoud, Julie Giraud, Xavier Gauthereau, Camille Blanchard, Sophie Daburon, Marco Zese, Silvia Molina-Castro, Pierre Dubus, Christine Varon, Helene Boeuf

**Affiliations:** 1 Univ. Bordeaux, INSERM, BIOTIS, U1026, F-33000 Bordeaux, France; aya.abou-hammoud@u-bordeaux.fr (A.A.H.); camille.blanchard@u-bordeaux.fr (C.B.); marcozese@hotmail.it (M.Z.); 2 Univ. Bordeaux, INSERM, BRIC-MIRCADE Team, U1312, F-33000 Bordeaux, France; 3 Univ. Bordeaux, INSERM, BRIC, U1312, F-33000 Bordeaux, France; julie.giraud@u-bordeaux.fr (J.G.); silvia.molinacastro@ucr.ac.cr (S.M.-C.); pierre.dubus@u-bordeaux.fr (P.D.); christine.varon@u-bordeaux.fr (C.V.); 4 Univ. Bordeaux, CNRS, ImmunoConcEpT, U5164, F-33000 Bordeaux, France; xgauthereau@immuconcept.org; 5 Univ. Bordeaux, IINS, U5297, F-33000 Bordeaux, France; sophie.daburon@u-bordeaux.fr

**Keywords:** secretome, conditioned media, paracrine, tumor, cancer stem cells, LIF, mESCs

## Abstract

Cancer stem cells are a subpopulation of tumor cells characterized by their ability to self-renew, induce tumors upon engraftment in animals and exhibit strong resistance to chemotherapy and radiotherapy. These cells exhibit numerous characteristics in common with embryonic stem cells, expressing some of their markers, typically absent in non-pathological adult differentiated cells. The aim of this study was to investigate the potential of conditioned media from cancer stem cells to modulate the fate of Leukemia Inhibitory Factor (LIF)-dependent murine embryonic stem cells (mESCs) as a way to obtain a direct readout of the secretome of cancer cells. A functional assay, “the StemDif sensor test”, was developed with two types of cancer stem cells derived from grade IV glioblastoma (adult and pediatric) or from gastric adenocarcinoma. We show that conditioned media from the selection of adult but not pediatric Glioma-Inducing Cells (GICs) maintain mESCs’ pluripotency in correlation with LIF secretion and activation of STAT3 protein. In contrast, conditioned media from gastric adenocarcinoma cells display LIF-independent stemness and differentiation activities on mESC. Our test stands out for its user-friendly procedures, affordability and straightforward output, positioning it as a pioneering tool for in-depth exploration of cancer stem cell secretome characteristics.

## 1. Introduction

Cancer stem cells (CSCs) are a subpopulation of tumor cells with self-renewal and asymmetric division properties. They give rise to more or less differentiated cells in the tumor mass. CSCs can be in a quiescent state in some conditions, resistant to conventional therapies and are at the origin of metastasis [1,2,3]. CSCs could survive when cultivated in 3D spheres on non-adherent tissue culture dishes without serum and with the presence of growth factors. In addition, CSCs can generate heterogeneous tumors after subcutaneous xenograft in immunodeficient mice [4].

CSCs have been characterized in solid tumors of various origins, including glioblastoma (GBM), the most aggressive and common type of primary brain tumor. GBM is known for its heterogeneity and lack of effective long-term therapy [5,6,7]. Although adult and pediatric GBMs are histologically indistinguishable, gene expression and mutational analysis have revealed many differences (e.g., IDH mutations, for example, confer an improved overall survival in adults with high-grade gliomas). Also, EGFR amplification is seen in up to 60% of adult GBMs, while its occurrence in pediatric high-grade tumors is less frequent. There is also a higher prevalence of histone mutations in pediatric high-grade gliomas [8,9]. Standard treatment for both the adult and pediatric GBMs includes surgical resection of maximal tissue and temozolomide therapy along with radiotherapy, but temozolomide chemotherapy seems not to be efficient in children. In the GBM model, detailed studies have been carried out to identify compounds which could ameliorate therapeutic approaches, like bisacodyl and the prozasin, both studied in adult Glioma-Initiating Cells (GICs), with potential interesting effects in reducing the tumor mass [10,11,12].

Additionally, CSCs have also been identified in gastric carcinoma (GC), which is one of the leading causes of death by cancer in the world. Chronic infection with *H. pylori* bacteria is responsible for more than 93% of non-cardia GC cases. Different classifications based on tumor histology and molecular analysis have led to characterization of diffuse, intestinal or intermediate phenotypes, from which various cell lines have been derived, named gastric cancer stem cells (GCSCs) [13]. These cancers, often detected too late, carry a poor prognosis [14,15].

Since a tumor communicates with its microenvironment, the characterization of conditioned media from tumor-derived CSCs could provide important insights into the most aggressive cells of the tumor and their impact on surrounding tissues. The secretome consists of proteins, growth factors, cytokines and other molecules secreted by cells which can have diverse effects on the tissue microenvironment. The secretome properties of tumor-derived cancer cell lines’ secretome could be revealed by a functional test based on the use of embryonic stem cells (ESCs). Indeed, these cells are an interesting receptacle of conditioned medium from CSCs, allowing characterization of stemness and/or differentiation properties of the secretome of any type of tumor cells [2,16,17].

ESCs are transiently pluripotent in vivo, and this state has been artificially captured in vitro using an appropriate culture medium, which differs between murine and human species. Indeed, murine ESCs (mESCs) rely on Leukemia Inhibitory Factor (LIF), while human ESCs and induced pluripotent stem cells (iPSCs) depend on basic fibroblast growth factor (bFGF) and Activin for pluripotency maintenance [18,19]. Interestingly, human LIF has higher affinity than murine LIF for the murine LIF receptor, making the mESCs model highly sensitive to any LIF activities present in human CSC conditioned media [20,21]. The LIF cytokine, which displays pleiotropic effects depending upon cell context, is secreted by tumor cells, but its precise function in the tumor context remains elusive. In some GBM-derived CSCs, LIF has been shown to have self-renewal activity, and blocking LIF signaling leads to tumor regression [22], but this effect is not general in GBM. However, similar functions of LIF have been described in chodorma (a rare tumor of spine and skull), in pancreatic tumors and in head and neck and lung carcinomas [23,24,25]. In contrast, in metastatic breast tumor cells and also in some pancreatic tumors, LIF is active as an anti-tumor cytokine in connection with the Hippo pathway [26,27,28]. Recently, we have also demonstrated a Hippo-dependent anti-proliferative and anti-metastatic effect of LIF in a subset of gastric-derived cancer stem cells [29].

Tumor development involves a dedifferentiation process which occurs with ectopic re-expression of stemness genes like Octamer-4 (*Oct4*), *Nanog*, Kruppel-like factor 4 (*Klf4*) or Kruppel-like factor 5 (*Klf5*) [30,31,32,33]. The common properties shared by many transforming events are that they target genes with key roles in early embryogenesis [2]. To investigate the embryonic-like properties of tumors, and in particular of tumor stem cells, the most aggressive part of the tumor responsible for chemotherapy tumor resistance and for the regrowth of tumor following chirurgical resection, we set up a functional assay named the “StemDif sensor test” on mESCs.

## 2. Materials and Methods

### 2.1. Cancer Stem Cells

GBM-derived cell lines, grown as neurospheres under specific growth conditions, have been previously described [6,10,34]. The status of these tumors (concerning IDH1/2 mutations and TP53 mutations) has been previously reported [35]. The OB1 cell line has been recently renamed TG1-C1 and is a subclone of the TG1 cell line, which has acquired the highest level of LIF, as shown in that study [10,12]. The adult GICs were grown in the NS34 medium, while the pediatric GICs were grown in the Nsah medium, previously described [34,35]

Gastric cancer stem cell lines have been previously described and were grown in the DMEM-F12 (Gibco, 11330032, Thermo Fisher Scientific, Bordeaux, France) (for AGS) or RPMI 1640 medium (Gibco, 11554516, Thermo Fisher Scientific, Bordeaux, France) for the other cell lines [29].

All media used to grow the different cancer cell lines behaved like that used for the mESC reference medium (DMEM + serum replacement medium (see below)) when supplemented with LIF.

### 2.2. LIF ELISA Test

LIF concentrations in conditioned media (CM) were determined by Enzyme-Linked Immuno *Assay* (ELISA) test, as previously set up [36]. Briefly, a Nunc Maxisorp 96-wells plate, flat bottom, was coated with 100 µL of the LIF primary antibody (1F10, monoclonal anti-human LIF AB, 10 µg/mL, [36,37]) diluted in coating buffer (carbonate buffer, PH 9.6: NaHCO_3_ 0.2 M/Na_2_CO_3_ 0.08 M/H20) overnight (O/N) at room temperature (RT). After two washes in PBST (PBS, 0.05% Tween), the plate was saturated with 200 µL/wells of PBS-bovine serum albumin (BSA) 1% for 1 h at RT. After two washes with PBST, the CM samples were added to the plate (100 µL per well) and incubated for 2 h at RT. Following incubation, the plate was washed twice with PBST (200µL per well). Then, 100 µL/wells of the 7D2-biotinylated LIF antibody (eBioscience, BMS1027 BIOTIN/C), Thermo Fisher Scientific, Bordeaux, France, 1 µg/mL in PBS/1% BSA, was added for 1 h at RT. After three washes with PBST, 100 µL/well of the streptavidine–peroxydase reagent (STDV-Perox, RPN1231VS, Amersham, Orsay, France) diluted 1:2000 in PBS-BSA 1% was added for 30 min at RT. After 2 washes in PBST, revelation was performed with citrate buffer: 100 µL/well of TMB (Sigma-Aldrich, T0440, Saint Quentin Fallavier, France), 10 min at RT, and the reaction was stopped by adding 50 µL/well of H_2_SO_4_ 1 N. The plate was read at 405/450 nm on a plate reader.

### 2.3. “StemDif Sensor Test”

mESCs (the CGR8 line) were cultured on plates coated with 0.2% gelatin (Sigma-Aldrich, G1393, Saint Quentin Fallavier, France) using DMEM-Glutamax high glucose (Gibco, 31966-021, Thermo Fisher Scientific, Bordeaux, France) with 10% serum replacement medium (Gibco, Thermo Fisher Scientific, Bordeaux France, 108280028), 20 ng/mL of human LIF (prepared in house from the CHO-LIF cell line, Refs. [36,38]) and 40 µg/mL of gentamycin (Gibco,, 15750-037, Thermo Fisher Scientific, Bordeaux, France). Cells were passaged twice a week, as previously described [39]. mESCs grown in the various media (NS34, Nsah, RPMI or DMEM-F12 without or with LIF) behaved as in the DMEM (SR medium used as the reference medium for expression-level data) (Figures S2 and S3).

For the “StemDif Sensor Test”, mESCs were washed once with PBS, and non-diluted CM were added for 5 days to cells. The medium was changed every two days until the analysis of morphology, alkaline phosphatase (ALP) activity (with the ALP kit: Sigma-Aldrich, 86R-1KT, Saint Quentin Fallavier, France) and gene expression or protein levels was performed.

For characterization of STAT3 activation (based on the presence of phosphor-Tyr-705 STAT3), mESCs were deprived of LIF for 24 h to turn off LIF signaling and incubated for 30 min with non-diluted conditioned media. Western blots of lysates from cells incubated with the reference medium without or with LIF were included as negative and positive controls, respectively.

### 2.4. RNA Preparation and Quantitative Real-Time PCR

RNA preparation and analysis were performed as previously described [39]. Briefly, cells on 6-well plates were directly lysed with 800 µL of TRIzol reagent (Life Technology, 15596026, Thermo Fisher Scientific, Bordeaux, France), and RNAs were prepared according to the manufacturer’s instructions. The reverse transcriptase (RT)/DNase step was performed with the QuantiTect Rev Transcription kit (Qiagen, 205311, Courtaboeuf, France). Quantitative real-time (q)-PCR was performed using the Applied Biosystems StepOneTM Real-Time PCR System CFX96 in a 25 µL reaction volume containing 5 µL of cDNA (1:20 dilution of the reverse-transcribed sample), 12.5 µL of B-R SYBR^®^ Green SuperMix for iQ (Quanta, BioSciences, VWR international, Fontenay-sous-bois, France, Premix Ex Taq (TAKRR420W)) and a primers pair at a final concentration of 0.5 µM. The PCR program included a denaturation step at 95 °C for 10 min, an amplification step for 40 cycles (15 s at 95 °C, 1 min at 60 °C) and a final dissociation curve step in order to determine the specificity of the product. Samples were duplicated for each run. Quantification was performed by calculating the 2^∆∆Ct^ value. Data were normalized using the murine Hprt mRNA, known to remain constant in the experimental conditions. The complete list of primers of pluripotent and differentiation markers used in that study is enclosed in Figure S1. The genes used as “stemness” or “differentiation” genes have previously been defined and characterized in extensive microarrays and functional studies performed in mESCs [16,40,41,42].

### 2.5. Protein Lysate Preparation

The cells were lysed in mild RIPA buffer containing the following components: PBS 1X, 1% Triton-X-100 (Sigma), 1% NP-40 (Sigma) and 0.05% SDS. Lysates were supplemented with a protease inhibitor cocktail (Sigma Aldrich, 8340, Saint Quentin Fallavier, France), 1 mM Pefabloc (Sigma Aldrich, 11429876001, Saint Quentin Fallavier, France) and Halt phosphatase inhibitor (Thermo Fisher Scientific, Bordeaux, France 78420). The lysates were then centrifuged for 20 min at 15,000× *g*. The antibodies used were diluted at a 1:1000 as follows: rabbit monoclonal anti-Phospho-tyr705 STAT3 (D3A7, Cell Signaling Technology, Leiden, The Netherlands); rabbit monoclonal anti-STAT3 (Abcam, Ab68153, Paris, France); rabbit polyclonal anti-Erk2 (Santa Cruz Biotechnology, sc-154, Clinisciences, Nanterre, France). The primary antibody incubation was performed overnight at 4 °C in a sealed plastic bag with slight agitation using the Odyssey blocking buffer (LI-COR Biotechnology, Bad Homburg, Germany). After washing with TBS 0.1% Tween (twice for 10 min), the membrane was incubated for 1 h with HRP-labeled secondary antibody: anti-goat IgG (H+L) (Vector Laboratories, Eurobio Scientific, Les Ulis, France), diluted 1:20,000 in Odyssey blocking buffer. After washing twice for 10 min with TBS 0.1% Tween and with TBS 1X, the membranes were revealed using the OdysseyFC (LI-COR) apparatus with the Image Studio, Odyssey 2.1 software, as recommended by the manufacturer.

### 2.6. Statistical Methods

Data were analyzed using one-way ANOVA with the Holm–Sidak test for multiple comparisons using the GraphPad PRISM Software v.10. The absence of significance, as observed for parts of the figures, came from the variation between data. However, the trend in gene expression values was in the same direction.

## 3. Results

**Figure 1:**
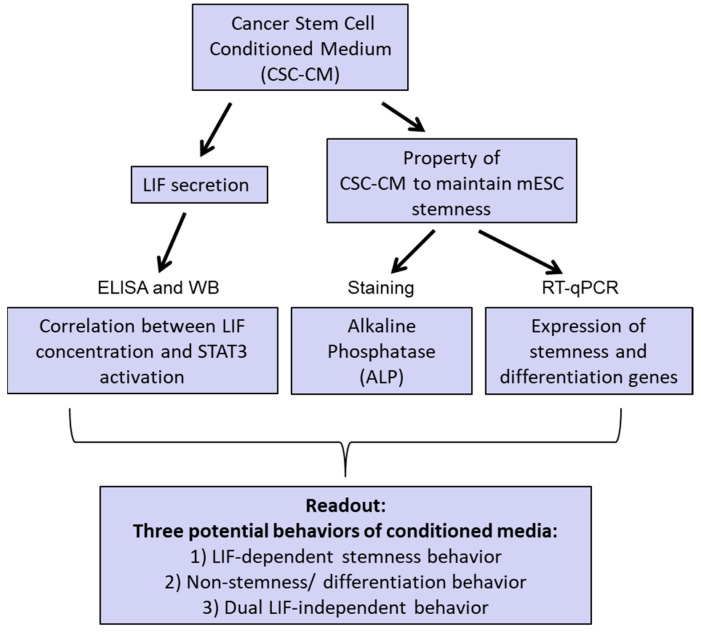
A new strategy to analyze the activities of conditioned media from tumor-derived cancer cell lines using a readout on mESCs, a LIF-dependent stem cell model.

The presence of LIF-dependent or any stemness activities in CSC conditioned medium could be an important parameter for deep characterization of tumors. We developed a functional assay based on the ability of conditioned media (CM) from tumor-derived cell lines to modulate mESC pluripotency, which is dependent on LIF. For each type of tumors, we assessed LIF secretion using an ELISA test and evaluated the impact of conditioned media on signal transduction and activation of transcription 3 (STAT3) activation, an essential LIF-dependent signaling molecule for pluripotency maintenance in the mESC model [39,43]. In addition, the effects of CM were tested on the morphology and gene expression profiles of mESCs. The schematic diagram shown in Figure 1 outlines our strategy.

### 3.1. LIF Secretion by Adult and Pediatric Glioma-Initiating Cells (GICs)

**Figure 2:**
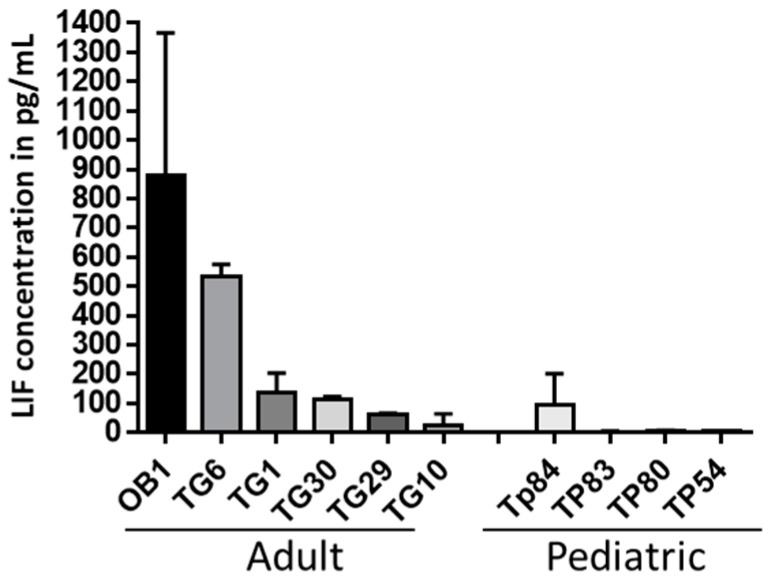
LIF secretion by adult and pediatric GIC (Glioma-Initiating Cell) lines. The graph represents the mean +/− standard deviation (SD) of LIF concentration of 2-day conditioned medium from adult or pediatric GICs, determined by ELISA test, n = 3 independent experiments.

We initially examined the secretion of the LIF cytokine in CM of a collection of human Glioma-Initiating Cells (GICs) derived from both adult and pediatric tumors using a LIF-specific ELISA test, as outlined in Materials and Methods. As shown in Figure 2, the results indicate that most adult GICs secrete LIF, in contrast to pediatric GICs, in which LIF secretion is absent except for one cell line (TP84).

### 3.2. LIF-Dependent Induction of STAT3 in mESCs Treated with GIC CM

**Figure 3:**
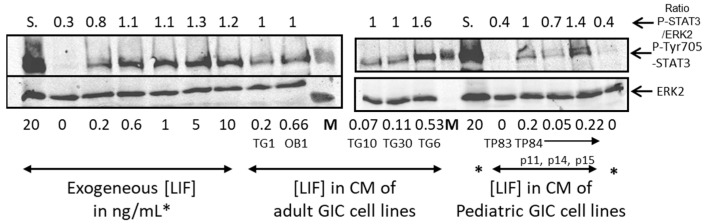
Correlation between LIF concentration in GIC conditioned medium (CM) and activation of STAT3. Western blots were performed on lysates of mESCs deprived of LIF for 24 h and then stimulated with GIC CM for 30 min. The blots were analyzed with the indicated antibodies. The concentration of LIF, determined by ELISA from the CM of each GIC, is expressed in ng/mL. CM from increasing passages (p) of the TP84 is shown. Lysates from mESCs treated with various concentrations of exogenous LIF are included as control for STAT3 activation. ERK2 is used as a loading control. The quantification of the ratio of Phospho-Tyr 705 STAT3 to ERK2 levels is indicated (Ratio P-STAT3/ERK2). S. indicates that the signal was saturated and therefore not quantified. The level of expression of total STAT3 protein was constant in these samples. M: molecular weight marker. *: exogeneous LIF in ng/ mL.

To determine whether LIF, secreted by GICs, is active and stimulates the Janus activated kinase (JAK)/STAT pathway, CM of each of the GICs were incubated for 30 min with mESCs that had been deprived of LIF for 24 h, a known condition for turning off LIF signaling [21,40]. The level of phospho-tyrosine 705 STAT3, a hallmark of active STAT3, was analyzed by Western blots, as depicted in Figure 3. Phosphorylation of STAT3 by various concentrations of human LIF (from 200 pg/mL up to 20 ng/mL) was included to directly compare the effect of LIF secreted by GICs with the LIF-containing medium, used to maintain mESCs in a pluripotent state. The concentration of LIF secreted by each tumor, determined by ELISA test, is also presented (Figure 3). We observed a direct correlation between LIF concentration and STAT3 activation, indicating that, in these tumor-derived cells, it is mainly LIF or a LIF-induced secreted factor that activates STAT3. For the TP84, the only pediatric GIC secreting LIF, we observed higher STAT3 phosphorylation with CM of the passages 11 and 15, in which LIF concentration is about 200 pg/mL. Interestingly we also noted a correlation between LIF concentration and STAT3 activation across different passages of this cell line. In conclusion, these findings shed crucial light on the dynamic interplay between LIF secretion and STAT3 activation, providing valuable insights into the regulatory mechanisms underlying stemness in tumor-derived cells.

### 3.3. “StemDif Sensor Test” Set up in mESCs

**Figure 4:**
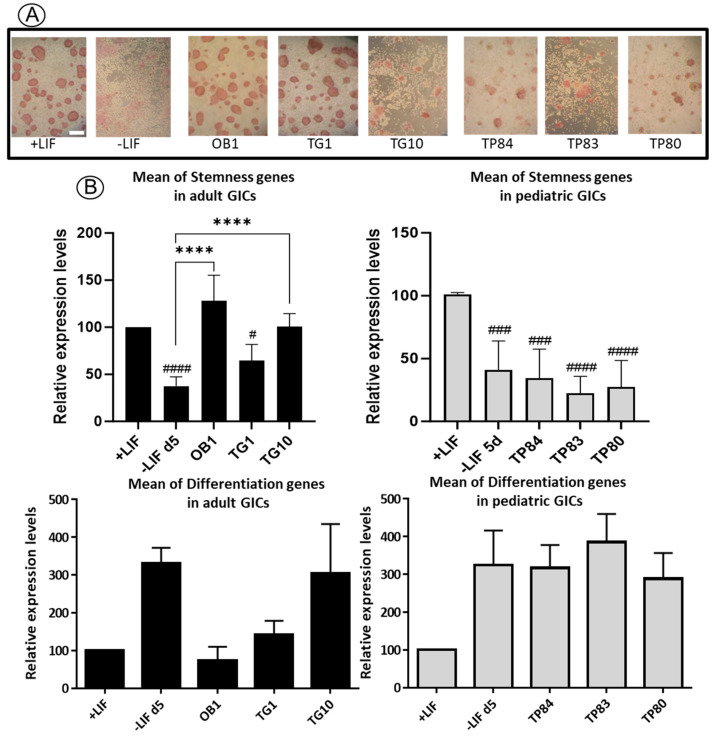
“StemDif Sensor Test”. (**A**) Pictures of representative mESCs stained to reveal active alkaline phosphatase (ALP+, clusters of red cells) after incubation for 5 days with conditioned medium (CM) of the different GIC lines as indicated. Scale bar is 100 µm. (**B**) Histograms representing the mean +/− SD of RNA expression levels of stemness or differentiation genes in mESCs treated for 5 days with CM of each adult or pediatric GIC cell line (2-day CM) as indicated. mESCs grown in the medium with LIF (+LIF, pluripotent condition) or without LIF (−LIF, differentiation condition) for 5 days were used as controls for mESC morphology and ALP staining as well as for mRNA expression studies by RTqPCR. The list of stemness and differentiation genes used for this study is included in Figure S1. Detailed experiments, from which the mean of stemness or differentiation gene expression were calculated, are shown in Figure S2. ^#^ *p* < 0.05, ^###^ *p* < 0.001 ^####^ *p* < 0.0001 vs. +LIF, **** *p* < 0.0001 vs.−LIF d5 in one-way ANOVA with Holm–Sidak test for multiple comparisons.

We then took advantage of the murine ESCs, known for maintaining pluripotency with elevated alkaline phosphatase (ALP) activity (red cells revealed by ALP kit) in LIF-containing medium, to test stemness and/or differentiation activities of the CM from a selection of GIC lines. Any stemness activity, where components are conserved between mice and humans (as is the case for the LIF ligand and receptors), could be characterized on mESCs. The mESCs were incubated with CM for 5 days. When mESCs were incubated with the adult GIC conditioned media, their morphology closely resembled that of ALP+ pluripotent mESCs, in contrast to mESCs incubated with pediatric GIC conditioned media (Figure 4A). Furthermore, the mean expression of a panel of pluripotent and differentiation markers was analyzed by RT-qPCR (Figure 4B). The expression profiles were compared to those of mESCs grown with (+LIF) or without (−LIF) LIF for 5 days, serving as controls for pluripotent and differentiated cells, respectively. The expression profiles of the markers analyzed were similar to those observed with LIF for mESCs incubated with adult GIC CM. In contrast, mESCs incubated with pediatric GIC CM displayed morphological differentiation along with expression profiles similar to the minus LIF condition (Figure 4A,B). This functional test revealed distinct behavior between adult and pediatric conditioned media. The selection of adult GIC-derived CM showed dependency on LIF for stemness activity, while the selection of pediatric GICs-derived CM did not secrete mESC-sensing stemness factors. This suggests the existence of additional pathways, distinct from those uncovered in the mESC model, that require identification. These pathways become activated and contribute to pediatric GICs’ tumoral properties. In summary, our findings underscore the critical role of LIF in maintaining stemness activity in adult CM, while highlighting the presence of unidentified pathways responsible for the tumoral properties observed in pediatric CM. This insight provides a valuable foundation for future research aimed at elucidating these distinct mechanisms and their potential therapeutic implications.

### 3.4. Gastric Adenocarcinoma-Derived Cell Lines Exhibit Heterogeneity in LIF Secretion, and Their CM Could Display Dual Functions on mESCs

**Figure 5:**
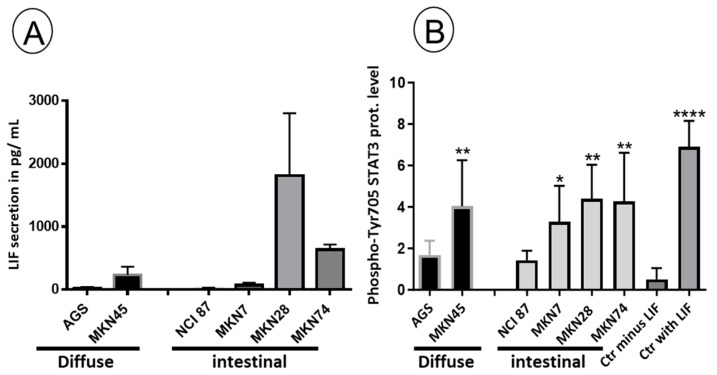
Gastric cancer cell lines secrete various levels of LIF, which induce phosphorylation of STAT3. (**A**) Graphs representing mean +/− standard error of the mean (SEM) of LIF secretion in pg/mL, determined by ELISA test in the different GC cell lines as indicated (in at least three independent experiments). (**B**) Histograms representing the mean +/− SD of the ratio of the level of phospho-Tyr705 STAT3/ERK2 (constant loading control), analyzed by Western blot in mESCs deprived of LIF for 24 h and stimulated with 2-day CM from each GC cell line for 30 min. The level of phospho-Tyr705 STAT3 in mESCs deprived of LIF for 24 h (Ctr minus LIF) and treated with 10 ng/mL of LIF for 30 min (Ctr with LIF) is also included, attesting to the effective LIF induction in mESCs. For (**B**): * *p* < 0.05, ** *p* < 0.01, **** *p* < 0.0001, in one-way ANOVA with Holm–Sidak test for multiple comparisons.

**Figure 6:**
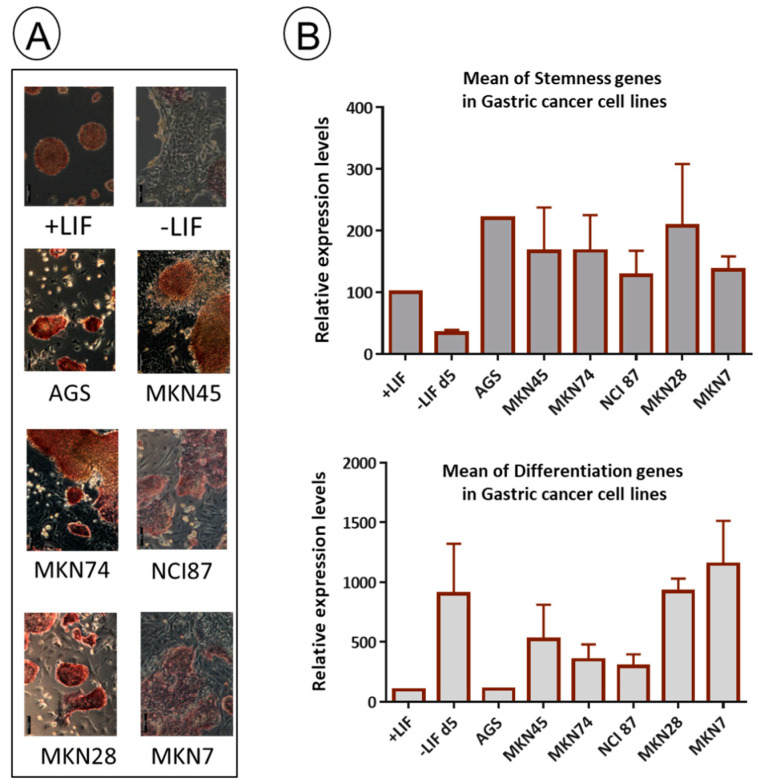
“StemDif Sensor Test”: (**A**) Pictures of representative mESCs stained to reveal active alkaline phosphatase (ALP+, clusters of red cells) after incubation for 5 days with CM of the different GC cell lines as indicated. Scale bar is 100 µM. (**B**) Histograms representing the mean +/− SD of RNA expression levels of stemness or differentiation genes in mESCs treated for 5 days with the CM of each GC cell line as indicated. mESCs grown in the medium with LIF (+LIF, pluripotent condition) or without LIF for 5 days (−LIF, differentiation condition) were used as controls for RNA expression, cell morphology and ALP staining. No statistically significant difference was observed in one-way ANOVA with Holm–Sidak test for multiple comparisons due to the variability in the expression levels of genes. The detailed experiments, from which the mean of stemness or differentiation gene expression was calculated, are shown in Figure S3.

We conducted an analysis of LIF secretion in GC cell lines previously characterized and classified as diffuse or intestinal tumor types [14,44]. As shown in Figure 5A, LIF is secreted at varying levels by the selected GC cell lines without any specific features associated with the tumor type. For example, the AGS cell line does not secrete LIF, while the MKN28 and MKN74 cell lines secrete a high level of LIF surpassing the LIF concentration required for cell signaling induction (around 100 pg/mL) (Figure 5A). Thus, we examined the potential of the GC conditioned media (CM) to activate STAT3 by analyzing the presence of phospho-tyr705 STAT3 in a test conducted as for GICs, shown in Figure 3. We found that all CM from the GC cell lines induced STAT3 phosphorylation, which is not strictly LIF-dependent (Figure 5B). Therefore, we conclude that there are LIF-independent activities present in the CM of some of the GC-derived cell lines which induce phosphorylation of STAT3. Next, we analyzed the stemness effects of the CM of these different GC cell lines on the mESCs. Remarkably, we found that all the tested CM, including those lacking LIF, induced stemness, as evidenced by a significant proportion of ALP^+^ undifferentiated cluster cells (Figure 6A). However, we also observed a proportion of mESCs that differentiated after incubation with the various CM. This indicates that mESCs respond to the complex composition of GC cell CMs. Furthermore, we characterized the gene expression of mESCs incubated with CM from various GC cell lines. We demonstrated that both pluripotency and differentiation markers are expressed by mESCs incubated with the different CM, with those from MKN28 and MKN7 exhibiting the best dual phenotype, while the one from AGS is the closest to pluripotent mESCs grown with LIF (Figure 6A,B). In conclusion, our comprehensive investigation highlights the intricate interplay between LIF secretion, STAT3 activation and the stemness properties induced by GC-derived cell lines. These findings underscore the multifaceted nature of the CM and its impact on mESC behavior.

## 4. Discussion

The re-expression or abnormal dysregulated expression level of stemness genes, like *oct4*, *sox2*, *klf4* and *klf5*, in differentiated cells are a common feature of the tumorigenesis process and have been well documented in many tumor types [45,46,47,48]. However, the presence of potential stemness and/or differentiation activities of the CSC secretome is much less explored. This knowledge could provide valuable insights into the impact of the tumor on its microenvironment. As a proof of concept, we developed a paracrine test named the “StemDif Sensor Test” on mESCs. This allows for the direct assessment of the presence of secreted stemness and/or differentiation activities conserved between mice and human, across various types of human tumors. Indeed, stemness or differentiation-inducing factors from CM could be easily traced in mESCs, by observing changes in the cell morphology and expression profiles of well-known pluripotent or early-differentiation mESC markers.

Among the cytokines secreted by various types of tumors that could regulate their status and potential metastatic issue is the LIF, a pleiotropic cytokine of the IL6 family which displays various effects on tumoral behavior. Indeed, depending upon which signaling pathway is activated (JAK-STAT or Hippo), LIF has been proposed as being pro- or anti-tumoral, as recently demonstrated in gastric cancer stem cell models [21,29,49]. Depletion of one subunit of the LIF receptor (LIFR), specifically gp190, has been found to be associated with increased metastatic processes in a breast cancer model in conjunction with a deficient Hippo pathway [26,27]. However, contradictory results remain concerning the impact of LIF in tumors [50], highlighting the importance of considering the effect of a particular cytokine or of a cocktail of secreted factors not only on the primary tumor but also on its microenvironment. As a potentially relevant receptacle for stemness and/or differentiation activities of the tumor secretome, we selected murine ESCs, which rely on the LIF cytokine for maintaining their undifferentiated state.

According to cytological markers, cell morphology and pluripotency/differentiation gene expression, we observed that mESCs respond differently to the complex composition of CM from different types of CSCs derived from the solid cancers studied, glioblastoma and gastric adenocarcinoma. We characterized three behaviors of conditioned media: a LIF-dependent stemness behavior (CM from adult GICs), a non-stemness behavior (CM from pediatric GICs) and a dual LIF-independent behavior characterized by the presence of both pluripotent and differentiated cells upon treatment with CM from gastric CSCs. Therefore, we demonstrated that mESCs can respond to both LIF-independent (as exemplified by the CM of the AGS cell line) and LIF-dependent stemness activities (as observed with CM of adult GICs). However, throughout this study, we observed variability in the results, which explains why we did not obtain statistical differences for some of the experiments, while the overall trend of the expression profiles was consistent.. For instance, gene expression closely ressembles that of stemness genes in the + LIF condition (eg: OB1 and TG1 for Figure 4B, in “Mean of differentiation genes in adult GICs”) while gene expression is close to that of differentiation genes in the −LIF d5 condition for Figure 4B, “Mean of differentiation genes in pediatric GICs”. Gene expression profiles in mESCs are known to be variable depending on cell confluency, on the potential of STAT3 or associated molecules to be properly activated and also in relation to the presence of low or high levels of feedback control proteins (e.g., suppressor of cytokine signaling 3 (Socs3) or protein inhibitor of activated STAT3 (Pias3), both of which are repressors of LIF/STAT3 pathway) [51,52].

An important finding of our work stands in the fact that secreted stemness activities, as characterized by functional tests in the mESCs model, do not necessarily correlate with tumor aggressiveness. While morphological and RNA studies from adult GICs and from gastric CM reveal stemness behavior and signatures, this was not the case with the CM of the selected pediatric GICs. Our finding raises questions about the association of secreted stemness signatures, at least those identified in the murine model, with CSC properties and impact on the surrounding tissues. It is evident that other stem cell models might be more suitable for testing CM of pediatric GICs, for example. However, given that human glioma CSCs are grown as tumorspheres in bFGF-containing medium, human ESCs or induced pluripotent stem cells (iPSC), which maintain pluripotency under bFGF-containing medium, cannot be used as receptacles for pediatric GIC CM [53,54]. In addition, an important parameter to take under consideration for the “StemDif Sensor Test” could be oxygen concentration. Indeed, hypoxic conditions, which will better mimic the stem cell niches, could provide a more accurate microenvironment for the characterization of CM behavior. Therefore, it is possible that the stemness activities of CM of pediatric GICs, ineffective in the mESC model under ambient air, could be revealed under hypoxic conditions. We could speculate that secretome activities are regulated under hypoxia, especially since we demonstrated, in the mESC model, that LIF activity was disturbed under low oxygen concentration [39]. It would also be insightful to characterize the impact of CM on Carcinoma-Associated Fibroblasts (CAF) in addition to the “StemDif Sensor Test” and to determine how these particular cells respond to the CM. Furthermore, this study should also be expanded to include more CSC models, encompassing various grades of specific tumor types. Therefore, our study paves the way for a more comprehensive exploration of the physiology of CSCs and of their secretome.

## Data Availability

Data is contained within the article.

## Supplementary Materials

The following supporting information can be downloaded at: https://www.mdpi.com/article/10.3390/biomedicines11123293/s1, Figure S1: List of primers used for RT-qPCR (murine sequences); Figure S2: Detailed results of RT-qPCR experiments used to build the graphs shown in Figure 4B; Figure S3: Detailed results of RT-qPCR experiments used to build the graphs shown in Figure 6B; Figure S4: A–D: Uncropped Western blots relative to Figure 3.
