# Supplementary material for: The “StemDif Sensor Test”: A Straightforward, Non-Invasive Assay to Characterize the Secreted Stemness and/or Differentiation Activities of Tumor-Derived Cancer Cell Lines
Source: Biomedicines. 2023 Dec 13;11(12):3293. doi: 10.3390/biomedicines11123293 (PMC10741605; doi:10.3390/biomedicines11123293)

Figure S1

List of primers used for RT-qPCR experiments, murine sequences

F: Forward: 5'

R: Reverse: 5'

			Size amplicon
**Reference gene:**			
nm_013556	Hprt F	TCAGTCAACGGGGGACATAAA	142
	Hprt R	GGGGCTGTACTGCTTAACCAG	
**Stemness genes**			
nm_013633	Oct4 F	GAGGAGTCCCAGGACATGAA	153
	Oct4 R	AGATGGTGGTCTGGCTGAAC	
nm_011443	Sox2 F	GCGGAGTGGAAACTTTTGTCC	156
	Sox2 R	GGGAAGCGTGTACTTATCCTTCT	
nm_028016	Nanog F	TCTTCCTGGTCCCCACAGTTT	100
	Nanog R	GCAAGAATAGTTCTCGGGATGAA	
nm_011934	Esrrb F	ACACTTGGGGACCAGATGAG	150
	Esrrb R	CCTACCAGGCGAGAGTGTTC	
X15351	Ceacam 1 F	ACTATCGTCGTACTCAGGCGA	98
	Ceacam 1 R	GTCACCCTCCACGGGATTG	
nm_008624	Mras F	TGTTCCAAGTGAAAACCTTCCC	117
	Mras R	GGGTCGTAGTCAGGCACAA	
nm_010637	Klf4 F	TATACATTCCGCCACAGCAG	136
	Klf4 R	TCTGGGCTTCCTTTGCTAAC	
nm_009769	Klf5 F	ACCTTACAGCATCAACATGAACG	123
	Klf5 R	TGGCTGAAAATGGTAACAGGTT	
nm_028679	Irak3 F	ACATGGGGCATCAACGAGC	231
	Irak3 R	GGAAGCTGATAGGGGTTTTCTG	
NC-000074	Rex1 F	CCCTCGACAGACTGACCCTAA	100
	Rex1 R	TCGGGGCTAATCTCACTTTCAT	
nm_010291	Gjb5 F	CCAAGCCCTCCGAGAAAAACA	107
	Gjb5 R	ACACCGCTTAATCACTAGGTAGA	
nm_023844	Jam2 F	GTGCCCACTTCTGTTATGACTG	113
	Jam2 R	TTCCCTAGCAAACTTGTGCCA	
nm_001042659	Fzd5 F	TCTTGTCTGCGTGCTACCTG	167
	Fzd5 R	GGCCATGCCAAAGAAATAGA	
nm_001160012	Gjb3 F	TGGTGGACCTACTTGTTTAGCC	155
	Gjb3 R	CTGGCGCACTGTACCAGAC	
nm_007657	CD9 F	GACTATGGCTCCGATTCGACT	101
	CD9 R	CCCGGCTCCAATCAGAATGT	
nm_010493	Icam1 F	GTGATGCTCAGGTATCCATCCA	213
	Icam1 R	CACAGTTCTCAAAGCACAGCG	

Differentiation genes

nm_010703	Lef1 F	AAGAAATGAGAGAGCGAATGTCGT	244
	Lef1 R	TTCTGGGACCTGTACCTGAAGT	
nm_007607	Car4 F	GACAACGGTTCAGAGCACAG	155
	Car4 R	AAGCCCTTGTTCACCTTGTC	
nm_016701	Nestin F	AGAATGTGCAGTCACCAAGG	138
	Nestin R	GGGTCTCATTTTCAGGTGGGT	
nm_011441	Sox17 F	GATGCGGGATACGCCAGTG	136
	Sox17 R	CCACCACCTCGCCTTTCAC	
nm_029357	Pcdh1 F	GCTCCGTATCTTCGAGTGGAT	97
	Pcdh1 R	GGATCTGGTTCTGGCATTCAC	
nm_011441	Brach F	GCTTCAAGGAGCTAACTAACGAG	117
	Brach R	CCAGCAAGAAAGAGTACATGGGC	
nm_010203	Fgf5 F	GAAAAGACAGGCCGAGAGTG	102
	Fgf5 R	GAAGTGGGTGGAGACGTGTT	
nm_008057	Fzd7 F	TGTGTCTCTCTTTCGCATCC	155
	Fzd7 R	GGAAGGCCTGCTCATAAAAG	

Figure S2 GENE Expression of mESCs with GICs CM.

Experiments 1 to 3 performed with the same CMs on independent experiments

Experiment 1

EXPRESSION of a selection of stemness and differentiation (Dif.) genes in different media:

SR: Serum replacement medium; NS34: medium adult GICs; Nsah: medium pediatric GICs

	**mras**	**ceacam1**	**esrrb**	**irak3**	**oct4**	**nanog**	**MEAN Stemness genes**
SR+LIF	100	100	100	100	100	100	100
SR-LIFd5	37	83	41	39	143	134	79
NS34+LIF	188	383	226	231	169	168	228
NS34-LIFd5	96	31	7	35	98	37	50
Nsah+LIF	419	896	192	578	147	517	458
Nsah-LIFd5	78	21	3	46	65	49	44

	**lef1**	**car4**	**pak1**	**fzd7**	**pdch1**	**Dif. genes**
SR+lif	100	100	100	100	100	100
SR-LIFd5	316	847	392	235	991	556
NS34+lif	235	300	129	146	373	237
NS34-LIFd5	533	1549	476	423	1547	905
Nsah+lif	241	566	152	174	615	350
Nsah-LIFd5	872	2194	483	408	1980	1187

EXPRESSION of a selection of stemness and differentiation genes in Adult GICs

	**mras**	**ceacam**	**esrrb**	**irak3**	**oct4**	**nanog**	**Stemness genes**
NS34+lif	100	100	100	100	100	100	100
NS34-LIFd5	51	8	3	15	58	22	26
OB1	100	48	72	49	68	226	94
TG1	73	68	78	52	71	131	79
TG10	66	133	90	79	92	200	110

	**lef1**	**car4**	**pak1**	**fzd7**	**pdch1**	**Dif. genes**
NS34+lif	100	100	100	100	100	100
NS34-LIFd5	227	516	369	289	415	363
OB1	3	73	43	34	20	34
TG1	108	137	102	121	74	109
TG10	160	373	124	125	125	182

EXPRESSION of a selection of stemness and differentiation genes in Pediatric GICs

	mras	ceacam	esrrb	irak3	oct4	nanog	Stemness genes
Nsah+lif	100	100	100	100	100	100	100
Nsah	19	2	2	8	44	9	14
TP84	6	21	6	14	51	39	23
TP83	4	4	12	5	64	10	16
TP80	7	7	6	12	70	8	18

	lef1	car4	pak1	fzd7	pdch1	Dif. genes
Nsah+lif	100	100	100	100	100	100
Nsah	362	387	318	234	322	325
TP84	319	648	101	8	316	279
TP83	147	325	110	185	122	178
TP80	317	379	132	77	139	209

Experiment 2

EXPRESSION of a selection of differentiation genes in Adult GICs

	lef1	car4	pak1	nestin	brach	fgf5	frzd7	pdch1	MEAN Dif. genes
NS34+lif	100	100	100	100	100	100	100	100	100
NS34	560	280	358	290	256	655	260	379	380
OB1	95	5	62	46	8	3	35	69	40
TG1	142	134	102	56	115	100	94	63	101
TG10	380	169	108	39	130	161	108	195	161

EXPRESSION of a selection of differentiation genes in Pediatric GICs

	lef1	car4	pak1	nestin	brach	fgf5	frzd7	pdch1	Dif. genes
Nsah+lif	100	100	100	100	100	100	100	100	100
Nsah	461	413	466	383	162	446	1607	589	566
TP84	234	86	47	204	28	633	8	141	173
TP83	439	179	141	403	116	629	1008	170	386
TP80	437	267	121	99	801	286	189	409	326

Experiment 3

EXPRESSION of a selection of stemness and differentiation (dif.) genes in different media:

SR: Serum replacement medium; NS34: medium for adult GICs; Nsah: medium for pediatric GICs

	gjb3	gjb5	jam2	fzd5	icam1	cd9	MEAN Stemness genes
SR+lif	100	100	100	100	100	100	100
SR	30	64	35	48	29	84	48
NS34+lif	184	218	206	140	80	207	173
NS34	27	82	35	209	17	166	89
Nsah+lif	314	1171	251	75	168	334	385
Nsah	20	126	28	178	16	183	92

	rex1	sox2	klf4	klf5	Stemness genes
SR+lif	100	100	100	100	100
SR	35	59	17	31	35
NS34+lif	167	151	177	179	169
NS34	9	152	55	34	62
Nsah+lif	114	119	257	476	242
Nsah	3	116	54	46	55

	gjb3	gjb5	jam2	fzd5	icam1	cd9	Stemness genes
NS34+lif	100	100	100	100	100	100	100
NS34	14	38	17	149	21	80	53
OB1	189	187	131	83	67	151	135
TG1	63	48	83	70	100	86	75
TG10	70	103	72	62	151	106	94

	rex1	sox2	klf4	klf5	Stemness genes
NS34+lif	100	100	100	100	100
NS34	5	101	31	19	39
OB1	116	91	170	77	113
TG1	82	87	54	71	73
TG10	90	84	83	93	87

	gjb3	gjb5	jam2	fzd5	icam1	cd9	Stemness genes
Nsah+lif	100	100	100	100	100	100	100
Nsah	7	11	11	236	9	55	55
TP84	11	21	16	14	4	32	16
TP83	6	3	9	73	12	19	20
TP80	4	16	3	10	12	29	12

	rex1	sox2	klf4	klf5	Stemness genes
Nsah+lif	100	100	100	100	100
Nsah	2	98	21	10	33
TP84	10	15	1	28	14
TP83	6	35	3	6	13
TP80	1	18	8	18	11

Experiments 4 and 5 performed with another set of CMs on independent experiments

Experiment 4

	oct4	nanog	klf4	ceacam	irak3	rex1	MEAN Stemness genes
SR+lif	100	100	100	100	100	100	100
SR-LIFd5	20	54	59	25	39	10	34
NS34+lif	23	171	164	45	138	39	97
NS34	1	15	147	9	35	2	35
Nsah+lif	43	204	177	44	112	68	108
Nsah	7	53	65	8	29	11	29

	oct4	nanog	klf4	ceacam	irak3	rex1	Stemness genes
SR+lif	100	100	100	100	100	100	100
SR-LIFd5	20	54	59	25	39	10	34
NS34+lif	23	171	164	45	138	39	97
NS34-LIFd5	1	15	147	9	35	2	35
OB1	36	467	128	135	143	91	167
TG1	15	75	44	19	49	20	37
TG10	28	211	135	44	99	27	91

	oct4	nanog	klf4	ceacam	irak3	rex1	Stemness genes
SR+lif	100	100	100	100	100	100	100
SR-LIFd5	20	54	59	25	39	10	34
Nsah+lif	43	204	177	44	112	68	108
Nsah-LIFd5	7	53	65	8	29	11	29
TP84	28	163	62	38	30	30	59
TP83	26	7	38	10	18	1	17
TP80	9	15	108	31	43	4	35

	nestin	brach	fdz7	pcdh1	fgf5	lef1	Dif. genes
**SR+lif**	100	100	100	100	100	100	100
**SR-LIFd5**	484	1383	376	350	799	95	581
NS34+lif	653	24	161	341	72	78	221
NS34-LIFd5	2510	12	267	242	29	169	538
Nsah+lif	354	16	118	142	189	42	143
Nsah-LIFd5	641	102	114	97	200	157	219

	nestin	brach	fdz7	pcdh1	fgf5	lef1	Dif. genes
**SR+lif**	100	100	100	100	100	100	100
**SR-LIFd5**	484	1383	376	350	799	95	581
NS34+lif	653	24	161	341	72	78	221
NS34-LIFd5	2510	12	267	242	29	169	538
OB1	74	22	43	680	52	8	146
TG1	439	54	194	197	305	108	216
TG10	1057	61	388	531	1237	129	567

	nestin	brach	fdz7	pcdh1	fgf5	lef1	Dif. genes
**SR+lif**	100	100	100	100	100	100	100
**SR-LIFd5**	484	1383	376	350	799	95	581
Nsah+lif	354	16	118	142	189	42	143
Nsah-LIFd5	641	102	114	97	200	157	219
TP84	1274	66	197	333	794	85	458
TP83	1746	123	190	397	528	134	520
TP80	1535	117	320	286	236	275	462

Experiment 5

	sox2	klf5	esrrb	mras	Stemness genes
**SR+lif**	100	100	100	100	100
**SR**	73	26	9	34	35
NS34+lif	126	84	102	187	125
NS34	233	71	4	199	127
Nsah+lif	123	76	114	103	104
Nsah	128	41	14	110	73

	sox2	klf5	esrrb	mras	Stemness genes
**SR+lif**	100	100	100	100	100
**SR**	73	26	9	34	35
OB1	95	100	166	164	131
TG1	85	40	25	88	59
TG10	188	96	80	114	120

	sox2	klf5	esrrb	mras	Stemness genes
**SR+lif**	100	100	100	100	100
**SR**	73	26	9	34	35
TP84	95	42	53	51	60
TP83	74	23	2	83	46
TP80	104	30	5	107	61

	sox2	klf5	esrrb	mras	Stemness genes
**SR+lif**	100	100	100	100	100
**SR**	73	26	9	34	35
NS34+lif	126	84	102	187	125
NS34	233	71	4	199	127
Nsah+lif	123	76	114	103	104
Nsah	128	41	14	110	73

	Fgf5	Lef1	Dif. genes
**SR+lif**	100	100	100
**SR**	293	43	168
NS34+lif	98	71	85
NS34	57	193	125
Nsah+lif	142	28	85
Nsah	199	143	171

	Fgf5	Lef1	Dif. genes
**SR+lif**	100	100	100
**SR**	293	43	168
OB1	64	7	35
TG1	371	121	246
TG10	1190	144	667

	Fgf5	Lef1	Dif. genes
**SR+lif**	100	100	100
**SR**	293	43	168
TP84	627	91	359
TP83	751	166	459
TP80	149	166	158

Figure S3 Gene expression of mESCs with Gastric CM

All experiments were done with independant CM of the various gastric cancer stem cells

Conditionned medium of Gastric cancer stem cells grown for 48h has been incubated with mESCs for 5 days

RNA prep analysed with various set of stemness or differentiated genes

Experiment 1

	oct4	nanog	ceacam1	klf4	irak3	rex1	klf5	sox2	esrrb	MEAN Stemness genes
SR+LIF	100	100	100	100	100	100	100	100	100	100
SR-LIF, d5	31	70	18	2	79	21	6	81	9	35
MNK45	105	140	67	67	88	95	32	152	51	89
MNK74	120	64	63	30	38	75	29	166	74	73
NCI 87	78	170	125	47	52	82	52	89	89	87

	lef1	nestin	brach	fdz7	pcdh1	sox17	Dif. genes
SR+LIF	100	100	100	100	100	100	100
SR-LIF, d5	42	160	370	30	156	53	135
MNK45	99	66	148	31	19	183	91
MNK74	451	43	233	52	363	305	241
NCI 87	685	569	155	187	248	537	397

Experiment 2

	oct4	nanog	ceacam1	klf4	irak3	rex1	Stemness genes
SR+lif	100	100	100	100	100	100	100
SR-LIF, d5	28	53	47	14	38	7	31
MKN7	69	265	25	50	131	27	94
MKN45	157	1082	307	286	200	160	365
MKN74	97	546	175	91	201	64	196

	nestin	brach	pcdh1	sox17	lef1	Dif. genes
SR+lif	100	100	100	100	100	100
SR-LIF, d5	533	1843	305	548	84	663
MKN7	428	1711	374	96	55	533
MKN45	120	45	575	176	13	186
MKN74	239	21	355	66	35	143

Experiment 3

	oct4	sox2	nanog	mras	ceacam1	esrrb	klf4	klf5	rex1	Stemness genes
SR+lif	100	100	100	100	100	100	100	100	100	100
SR-LIF, d5	108	57	60	13	24	7	11	79	2	40
MKN7	106	104	86	36	70	369	81	192	24	119
MKN28	198	225	223	213	334	708	129	310	102	271
MKN45	87	22	22	18	46	47	5	89	23	40
MKN74	91	38	47	71	145	76	26	105	89	77

	pcdh1	nestin	brach	sox17	lef1	Dif. genes
SR+lif	100	100	100	100	100	100
SR-LIF, d5	1180	3558	5541	97	386	2152
MKN7	870	925	751	203	2900	1130
MKN45	256	711	663	35	651	463
MKN28	720	667	1488	311	1966	1030
MKN74	63	190	240	59	862	283

Experiment 4

	oct4	nanog	sox2	rex1	mras	klf4	klf5	ceacam1	esrrb	Stemness genes
SR+lif	100	100	100	100	100	100	100	100	100	100
SR-LIF, d5	16	19	20	6	10	13	27	13	3	14
AGS	90	377	264	169	101	305	201	150	318	220
NCI 87	94	277	155	115	226	143	97	265	130	167

	lef1	pcdh1	nestin	brach	Dif. genes
SR+lif	100	100	100	100	100
SR-LIF, d5	23	105	90	107	81
AGS	23	23	157	215	105
NCI 87	67	227	280	239	203

Experiment 5

	Oct4	Sox2	Nanog	Ceacam1	Esrrb	Mras	Klf4	Klf5	Rex1	Stemness genes
SR+lif	100	100	100	100	100	100	100	100	100	100
SR-LIF, d5	72	84	25	47	33	56	43	23	59	49
MKN7	207	167	116	190	143	241	175	119	148	167
MKN45	210	192	164	236	202	134	138	106	129	168
MKN28	79	51	44	225	88	192	176	27	70	106
MKN74	85	133	120	223	217	890	754	311	146	320

	Lef1	Brach	Car4	Nestin	Sox17	Dif. genes
SR+lif	100	100	100	100	100	100
SR-LIF, d5	863	933	520	902	341	712
MKN7	487	6730	404	1136	200	1791
MKN45	684	1469	2052	1233	1352	1358
MKN28	90	2193	302	1328	135	810
MKN74	116	347	697	1936	575	734

Figure S4: Uncropped western blots corresponding to Figure 3.

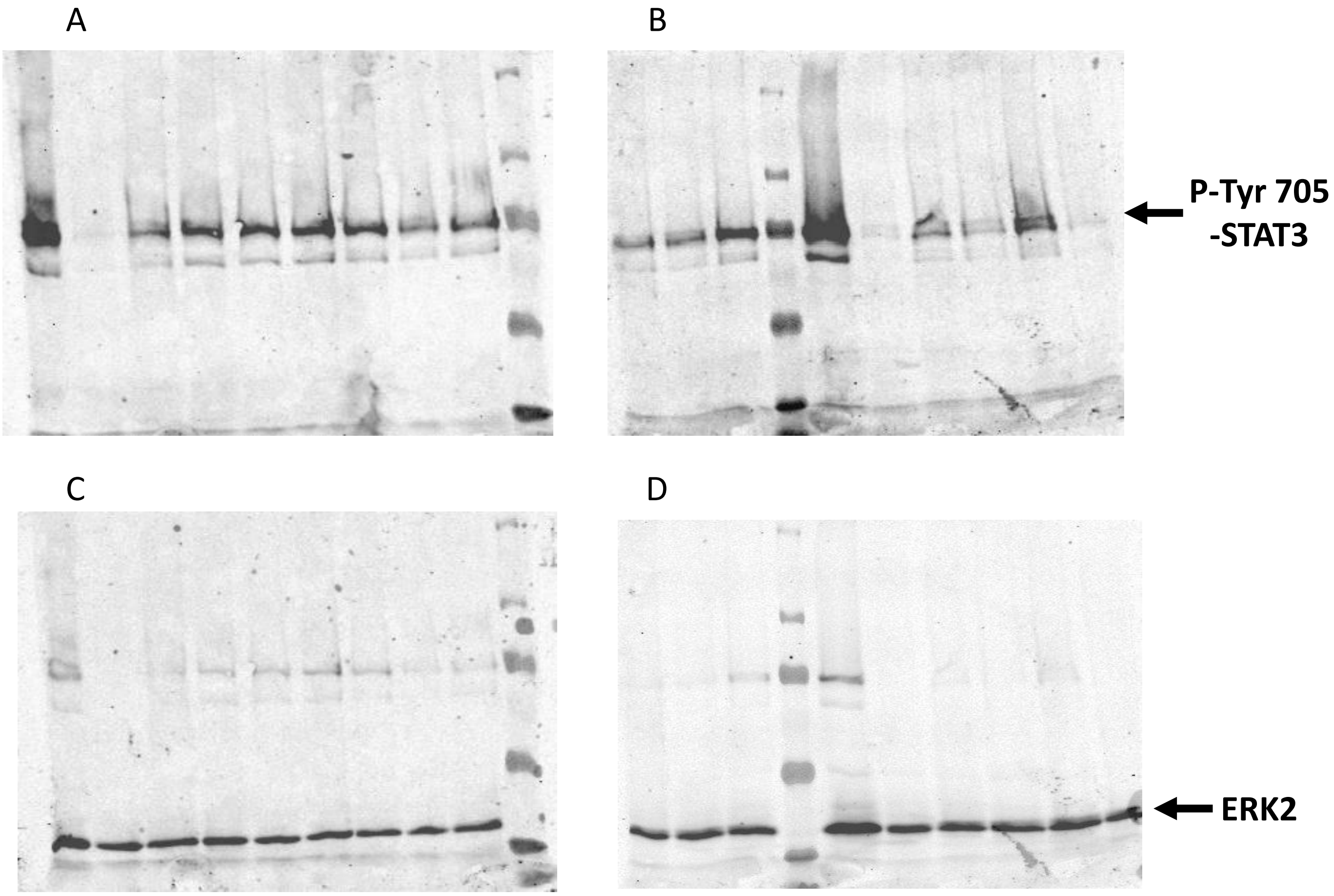

Supplement: Supplementary file 1 [file biomedicines-11-03293-s001.zip › biomedicines-2692672-supplementary.pdf]
